# A Friendly Alias

**Published:** 1887-07-16

**Authors:** 


### A Friendly Alias.

By W. J. E.

### Chapter I.

"The crisis must be past. It seems an age now since I received Willie's letter. Yet it was only last evening's post. And since, have I been awake for thirty hours? I don't know. I went to bed up into this little room, earlier than usual, before the rest. But sleep? That could not be sleep. The pillow was damp this morning; my eyes were so red, I was ashamed to go down; and I distinctly remember opening my eyes time after time through the long night, wishing it were morning. And when morning came I was utterly weary and ill. Now the day has passed somehow, I have seen and heard, eaten and drunk, but all mechanically. I have taken no interest in anything. Bearing with me a dull pain at heart and a throbbing head, I have gone to and fro in the house, answering Tom and Elsie and mamma, and all the time whispered to myself, with sigh after sigh, 'that letter!' Yet collect my thoughts I could not. I have felt as one having no memory, just gazing and gazing at some dull glare of flame, without being able to close his eyes.

"To-night I have crept away again and sat looking out of this window before which I am writing, down the brae on to the winding river. I looked westward up the river towards St. Columban's old cathedral, whose twin towers rose darkly and clearly like silhouettes against the pale silvery green and blue of the late sunset. I have often sat here before thinking of Willie. Often and often we have sat among the trees at the river's edge, and watched its waters, often walked up the other side, the Craigdalloch side, past where the river makes that bend and comes leaping, bubbling, and spray-white over the boulders which lie across its way. Every tree and stone, every landmark has some association with Willie, and seems, now he is gone, a friend and confidant.

"As I looked I became more soothed, and began to collect my thoughts. I did not feel as if I could sleep, but just sit and sit until—until morning, I suppose. Up here in the north of Scotland in summer there is no real darkness at night. The long-drawn semicircles of changing sunset light have not entirely faded away in the west before the dark night-clouds over the sea to the east begin to furl up and disclose the first red flushes of sunrise.

"As I sat and grew calm, it came into my head to write down some of my thoughts in the book Willie gave me. He said it would sometimes amuse me to write in it, not, as in some girls' diaries, the commonplace events which need no recording, but thoughts and imaginings as they occurred to me, or a deep impression was made by events upon my mind. Amuse me to write and read! Yes! it has done. It has often been to me instead of him. I little thought I should ever be writing in it, not for amusement, but sadly, slowly, to collect my scattered, stunned senses, and tire myself to sleep.

"For some time Willie's letters had been few and far between; shorter, less full of those little bits of personal news which I like so much, less full of his old cheerfulness. Ever since that great misfortune came upon him—poor Willie—when those wretches of solicitors failed and defrauded their clients of their all, there has been a great change in him. But why should the change have been so great? He is clever and steady and I have told him many a time in my letters that what was mine was his. Always he has replied that he must make his way himself, must not rely upon other people for his living, must struggle; and hard, hard work it was, to get even on to the lowest rung of the ladder.

"Then grandmama died—poor darling grandmama! How good she was to us all, and most of all to me! What long talks we had together of Willie. She longed to see us married, for she had taken quite a fancy to him, critical as she was about most people. When she died, and her will was opened, it was found that she had left Willie three thousand pounds on the condition that he married poor little me. I thought no harm of it. It seemed a very last piece of grandmama's kindness, and it was so meant, I'm sure. I wrote and told Willie, and, knowing his proud temper, only hinted at the condition, but never dreamed of offending him. Then last night came his letter, just when I thought my difficulties were clearing away. Let me read it again. I scarcely need. Every word is seared into my brain as with a hot stile,—

"'Dear Christina,'—(the very beginning made me feel a tremor—he never called me that—I was always Chris or Kirsty),—'I can no longer refrain from writing plainly. I have given you hints, but you do not or will not see them. Can you not realise how I am placed, that I am practically penniless, and living from hand to mouth, that I have no settled means of livelihood? But how should you, nursed, reared, and still living in affluence?

"'The thought of the cup of happiness once so near my lips, now dashed to the ground by the failure of those rascally scoundrels, and of my own foolishness in not looking after them, unnerves me. The thought of you cherishing vain hopes, and wasting your young years and fresh beauty waiting on a delusive chance, is one that makes me feel myself your wrong-doer, and undermines my energies and the possibilities of achievement.

"'You know I will not accept a share of your portion till I can offer you a home, and at least a competence without it. Now your relatives hold out to me a bribe of three thousand pounds to become a beggar *protégé* of the family, to be an heiress's husband. No! I have still a few brands of pride of blood aflame within me. I have still some powers of mind which, untrammelled, will raise me to a self-won position. Then, and not till then, may I indulge in ideas of marriage. I ask you to release me from my promise. You can lose nothing by severing the links uniting you to a poor man. You have everything to gain by freedom.

"'Yours faithfully,

"'W. LANGLEY HEATHCOTE.'

"I think I have read somewhere that love is in a man's life a mere incident, taking its turn with business, reading, and other occupations, but in woman it is her whole life. What it may be with other women I don't know. I cannot think that those who so easily get engaged and as easily break off the engagement know what love is. I am sure few men realise it to the full. Willie asks me to give mine up, tells me I lose nothing. Oh! Willie! Willie! How am I to answer you? Have my letters shown nothing? Have my spoken words, my very looks in your company told you nothing? Yet you ask me to give up what has been the sole object of my existence since I knew you, the darling hope of being some day your wife. I cannot bear to face the thought of the ordinary dull round of a woman's life without it, of having this 'unharvested excess of love' stored up and mouldering in the garners of my heart until it becomes soured and harmful from lack of use. To-night,—it is no longer night,—there from the other window, I see shining over the sea that early streak of white light that makes some of the wavelets glitter while the rest are grey. My eyes are aching. I must lock your clasp and lay you aside, my only confidante, and try to sleep. Willie, I cannot bear to answer you. To-night!"

### CHAPTER II.

SOME nights after the entry in a lady's hand of the monograph just set down, two young men were striding vigorously along the quayside at Newcastle-upon-Tyne. The night was dark. Black, angry-looking clouds were plentiful, and the appearances of the moon from behind them very few. Along the quay it was much darker than in the main streets. The tall warehouses and offices, mostly unoccupied and unlit at night, the narrow and dark openings up courts, and "chares," and stairs, the uncertain glimmer of an occasional gas-lamp or ship's lantern, casting a ray now and again on piles of landed goods which in places strewed the quay, all contributed to make it an unsafe, or at least hazardous promenade. Yet to anyone with a turn for the picturesque or for the sombre, there was something especially attractive in the darkened shadowy way, in the gables and projecting upper-stories of the older houses, which alternated with the huge modern structures, standing out against the sky, in the ghost-like masts and creaking cordage of the shipping (for every respectable ghost should possess a creak). Last of all there was infinite attraction in the untiring, turbid, and murky waters of the Tyne, rushing, eddying, swirling past the bridge and round about projecting landing-stages and anchored vessels, finally making up their minds to go forward, as became a steady old mercantile river long past its youth.

To anyone tired of the noise and glare of Grainger or St. Nicholas Street, it was a great relief to walk along the quay, and it was with some half-idea of soothing his friend's agitation that Cyril Clayton had directed his steps that way. It had been a favourite stroll of theirs when enjoying an evening's pipe. But to-night Langley Heathcote was too troubled to smoke. Nothing but hard walking or rapid action would serve. At such a time some men will drink hard, men who drink little at ordinary times, and a quarrel arising out of drink is welcomed and pursued with a bitter zest altogether disproportioned to its cause: a result of the original excitement.

Cyril, accustomed to his friend's moods, had perceived that Heathcote was perturbed, but had left it to him to set the pace and to open his mouth when it pleased him. Confidence, well grounded on a long acquaintance and many a mutual service done in days gone by, was entire between these two men. There was no need to ask questions. And presently Heathcote made a remark with a vindictive earnestness:

"I wish all the shipping-offices, brokers, agents, and all were at the bottom of the river!"

"Thanks, old man; and where should I be? Also tasting the mud?" retorted Cyril with a laugh.

"Don't chaff; I can't stand it," returned the other, but not so angrily. "You know what I mean."

"What's the particular animosity just now? What's the shipping interest done now?"

"Perhaps my luck is to blame," Heathcote went on. "Here these two or three weeks have I been running up and down office-stairs interviewing quay potentates, the cynosure of smooth-chinned clerks and pupils in the outer offices; kept gazing by the hour at section-models of ships, charts, and ledgers; subjected to curt examinations in Mr. So-and-So's private room, and all to no purpose. Day after day has passed, and no appointment got, or apparently any chance of one."

"Yet you can't blame them, you know," said Clayton, persuasively. "With these men—and I suppose I am a poor specimen of them—all their money-making depends on their own shrewdness and on using the cheapest, provided they are effective, methods. We have a certain undescribed standard for the effective clerical machine, just as we have a standard boiler or cylinder, or steering gear. We want a man who knows every office on the quay or in the city, used to handling books, rapid and accurate at figures, and up in a lot of general knowledge related to shipping. A very few minutes would suffice for one of these Tynesiders to find you fell short of the standard, you a man of University education, gentlemanly address, and not a vestige of commercial experience."

"Experience!" echoed Heathcote bitterly, playing with the word. "This fortnight has been 'commercial experience' in all conscience. It seems to me that one is always gaining fresh experience, but the occasions for using it do not occur again. When shall I again have the chance of losing a fortune or keeping it, by trusting or distrusting family solicitors? No one guides himself by other people's experience or stays to listen to it."

"That's only true to a certain extent," said Cyril. "But, for all that, I believe with Garfield, that where a man is thrown into the sea of life to sink or swim, he'll swim in the end if there's anything in him. And it's a good thing for you, bent as you are on relying on your own resources, and not on floating corks, to realise at the ouset the roughness of the wave, and the useless ness of kid-gloves as a life-preserver."

"You're right, old man," said Heathcote in a tone of conviction. "I mean to swim somehow, even if it does come to breaking stones or cab-driving. In the mean-time I have taken a decisive step in regard to . . ."

"Miss Seaton?" interposed Cyril, in a tone of respect-

"Yes! One which partly ends my terrible anxiety on that subject."

"How do you mean?" Cyril asked.

"You remember me telling you I wanted to close the engagement. It was made at a time before the crash came, when I stood on the green, smiling surface of the volcano before the crater burst out, when my position and Kirsty's were equal. But now how could I hold her to her promise, how fulfil mine? She is unfit for a life of pinching economy, a girl brought up to shine in society, not in the kitchen. Dear, affectionate girl that she is, in all innocence of hard facts, sitting on a cushioned chair, writing on scented notepaper, she can say that she would willingly work for me, bear any privation for my sake and in my company."

"And so she should, if she is the real true girl I should want—but then, I know nothing about them!" was Cyril's remark.

"That's all very well," Heathcote went on, "but when the first few months of married life have passed, when what the girl considered necessaries the wife must forego, then the word of reproach is spoken, or the look that speaks louder than the word. Then come the 'why did I marry you? How comfortable I was at home!' and other imaginable stinging phrases."

"But she has money?" Cyril asked, as he put his hand in his pocket for his latchkey. They had stopped at the door of a handsome house in Jesmond. "You'd better come in and take some coffee," he added, seeing Heathcote hung back.

"Yes!" said Heathcote, as they entered, "I never inquired how much, but there my pride comes in. I could never bring myself to live on my wife, however much I loved her. People never give you credit for disinterested affection in a match like that."

They were now inside a luxuriously furnished sitting-room, which bore all the traces of its bachelor owner-ship. Clayton went on, as he began to fill a pipe:

"All men would not be so particular. But you said you had taken a step'?"

"Yes!" said Heathcote, in an almost defiant tone, as if he expected reproof. "I have closed the engage-ment."

Cyril lit his pipe, and then repeated in astonishment —"Closed the engagement. After so long intimacy?"

"Yes! I was oppressed by my non-success, my anxiety for our joint futures, by my very love for her. I resolved to separate our fortunes. I wrote and asked for my release. That is a copy of my letter," and he handed Cyril the draft of what we have seen. It was almost without correction, and bore traces of being written hurriedly, as if the thoughts sped too fast for the pen.

Cyril read it carefully, so carefully that he stopped smoking and his pipe went out. Then he returned it, saying:

"You know I don't go in for these things myself never did, but I should say that letter is too hard. You wrote it in a fierce mood. The letter reflects it. It must have wounded her feelings much more than was necessary in accomplishing its object. And it sets your character in a worse light than it deserves. I see no bribe in the legacy you spoke of. I see nothing but a kindly old lady wishing to make her granddaughter comfortable, and at the same time show her approval of that granddaughter's choice. I can appreciate your true motives for wishing to be free, and to give her freedom while she is able to take advantage of it. But, if your past estimate of her is true, girls like her do not lay down their love easily, if they do their engagement ring. There must be a wrench which neither you nor I can appreciate."

"You are getting quite dogmatical," Heathcote inter-posed, "even if you have had no experience."

"That is how I would have women be," Clayton went on, not noticing the sarcasm; "if they are not. But at present I shall believe it. You never ought to have written such a letter. Has she replied?"

Heathcote took out of a pocket-book, and his voice was husky as he offered to his friend a letter in a feminine hand, saying, "Read for yourself!"

Cryil protested against it. But Heathcote insisted, and he read:

"Gordon Brae, Strathhaven.

"My dear Willie,—I could not find heart to answer your letter before. I have been wretched and ill since it came. It is hard, oh! so hard, Willie, to have to give up hopes I have cherished so long that they have become part of my very being, ever present to my mind, ever the greater half of my happiness. So that I might one day be your wife, I did not mind waiting; I have told you often. I did not mind working. No woman is fit wife who cannot share her husband's toils and troubles as well as his joys. I can only say now I would have done it unmurmuringly. And even now, out of the great love I bear you, I will obey you. Your wish is my will if it is for your good. I will not be a clog on your endeavours, not for an hour. If the release from your engagement will set free greater energies to meet your difficulties, I no longer hold you bound. How I shall bear it, Willie, I do not know. I dare not think. There is One who will help me who once suffered keener griefs.

"I send back our engagement ring. I will send back all your other gifts and your letters if you wish, but I am jealous of parting with a single memorial of you. I shall never marry anyone else. I have once loved; I still love. My hand goes not without my heart, and that can never change.

"You will not let me be your wife, Willie. You can still let me be your sister. Let me write to you some-times. Keep me always informed of your address, and, as due to an old acquaintance, do write to me now and again.

"I cannot write more now. God bless you, my dearest Willie, and give me strength to bear this severance.—Ever your loving friend,

"Christina Seaton."

(*To be continued.*)

